# Analysis of Latent Esophageal Perforation Caused by a Mysteriously Migrated Anterior Cervical Plate Into the Gastrointestinal Tract

**DOI:** 10.7759/cureus.99272

**Published:** 2025-12-15

**Authors:** Harun E Sen, Omer Gunal, Erkan Kaptanoglu, Volkan Etus

**Affiliations:** 1 Neurological Surgery, Kocaeli University, School of Medicine, Kocaeli, TUR; 2 General Surgery, Marmara University, School of Medicine, Istanbul, TUR; 3 Neurological Surgery, Okan University, School of Medicine, Istanbul, TUR

**Keywords:** anterior cervical spine surgery, complication, conservative treatment, esophageal perforation, implant migration

## Abstract

Anterior cervical implant migration through the pharyngoesophageal perforation is a very rare but serious condition that often necessitates surgical intervention. Nevertheless, rare cases have demonstrated recovery through conservative management. This article presents a 40-year-old man with dislodgement of an anterior cervical plate five years post-surgery. A last-minute radiography prior to salvage surgery revealed that the implant was no longer in its original position, and subsequent imaging traced its journey through the gastrointestinal tract. The esophageal perforation caused by the migrated implant was managed with a conservative approach. This study reviewed the literature of disappeared or naturally displaced cervical implants that resolved without surgery. An implant's disappearance or natural excretion is rare, with clinical presentations ranging from asymptomatic cases to severe symptoms such as dysphagia or oral expulsion. The time interval between surgery and implant excretion varies from five weeks to 11 years. Asymptomatic gastrointestinal elimination is more frequent in men, whereas oral expulsion predominates in women. Although younger patients generally recover faster, conservative management is also effective in older individuals. This study emphasizes the importance of last-minute radiological imaging prior to surgery in patients with implant dislodgement, and a "watch and wait" conservative approach should be considered in certain esophageal perforation cases. Additionally, a missing hardware could be considered to have been excreted through the gastrointestinal tract.

## Introduction

Anterior cervical spine surgery has been a widely utilized and refined approach for the treatment of cervical myelopathy, degenerative disc disease, neoplasms, spondylosis, and traumatic injuries over the past half-century [1-4]. Despite its widespread adoption and the proficiency achieved in its execution, the procedure is still associated with various potential complications [1,5-7]. Although the extrusion of implanted instrumentation resulting in esophageal perforation is an extremely rare complication, it is among the most severe and least desirable outcomes, given its high mortality rate [4,7-9]. It needs complex surgical interventions and prolonged hospitalization. In rare cases, this complication can be unexpectedly manageable. Notably, a conservative approach, typically employed for small esophageal perforations with mild clinical presentations, may also be effective in cases caused by implant penetration, as is our case. This observation is both intriguing and rarely documented in the literature [2,3,9-22]. 

This study details a case of an anterior cervical plate and screws dislodging five years after surgery, leading to an esophageal perforation that was effectively managed with conservative treatment. The implant's passage through the gastrointestinal tract was thoroughly documented radiologically. Additionally, we present a review of cases in the literature where pharyngoesophageal perforations caused by implant extrusion were managed without surgical intervention.

## Case presentation

**Figure 1:**
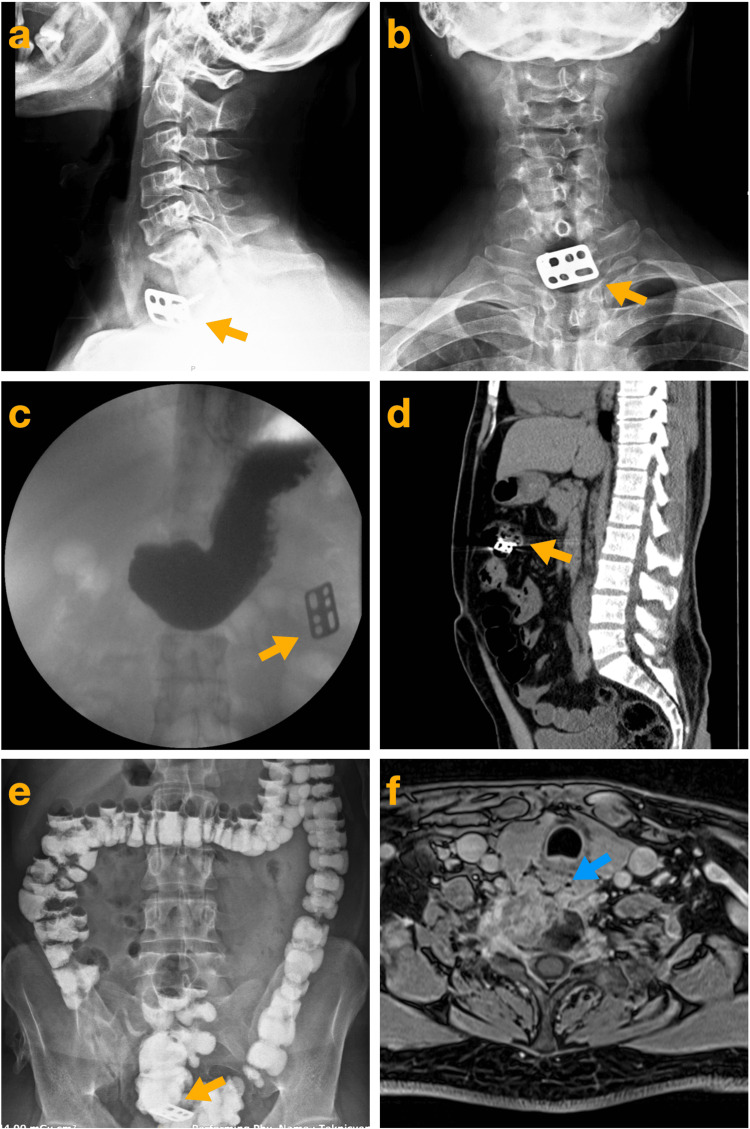
Imagings of the implant's course in the gastrointestinal tract Lateral (a) and anterior-to-posterior (b) radiographs demonstrate the dislodged plate (yellow arrows) attached to the vertebral body with a single screw. Contrast esophagography reveals the plate's location (yellow arrow) in the abdomen, at the level of the gastric (c). Sagittal computed tomography imaging shows the plate (yellow arrow) in the transverse colon (d). Contrast colon radiography illustrates the migration of the plate (yellow arrow) into the sigmoid colon (e). Contrast magnetic resonance imaging reveals abscess formation in the right anterior paravertebral area adjacent to the esophagus (blue arrow) (f).

A 40-year-old male patient presented to the clinic with dysphagia that had persisted for approximately one month and was gradually worsening. Five years earlier, he had undergone anterior cervical spinal fusion (C6-T1 anterior cervical plate) at another institution due to post-traumatic dislocation. Recently, he experienced occasional throat pain, exacerbated by movement and dysphagia, although the discomfort was not severe. Cervical imaging revealed that the plate was dislodged and the screws were missing, with only one screw visible (Figure 1a and 1b). The patient was informed of the dislodged implant, and surgery was recommended.

The patient was admitted for surgery three weeks after the diagnosis, which was delayed due to patient-related factors. During this period, he did not experience worsening dysphagia, fever, severe pain, or upper airway obstruction. Preoperative hematologic evaluation demonstrated elevated inflammatory markers, including an erythrocyte sedimentation rate of 38 mm/h (reference range: 0-30 mm/h), a C-reactive protein level of 13 mg/L (reference range: 0-5 mg/L), and a white blood cell count of 11.2 × 10⁹/L (reference range: 3.46-10.04 × 10⁹/L), despite the absence of any clinical signs of infection. A radiograph taken on the day of surgery confirmed that the dislodged plate was absent from its original position. Contrast esophagography revealed a minimal leak and showed that the dislodged plate had migrated into the abdomen at the level of the stomach (Figure 1c).

Given the patient's stable condition, the decision was made to postpone surgery. The cessation of oral intake was maintained, and broad-spectrum antibiotics were initiated. A computed tomography (CT) scan of the abdomen confirmed that the plate was located in the transverse colon (Figure 1d). The following contrast colon radiography demonstrated that the plate had migrated into the sigmoid colon (Figure 1e). On the fourth day, radiography showed that the implant was no longer visible. The patient passed the implant asymptomatically, though it could not be retrieved from the stool. Magnetic resonance imaging revealed the presence of a cervical paravertebral abscess neighbouring the cervical esophagus (Figure 1f). Cervical flexion-extension radiography showed no signs of instability. Following the migration of the implant through the gastrointestinal tract, the patient's symptoms improved significantly, and his overall condition remained stable. Upon this improvement, surgery was not pursued, and conservative treatment continued. The patient was discharged after two weeks uneventfully. Antibiotic therapy was maintained for six weeks until infection markers returned to normal.

## Discussion

This study involved a review of the English literature reporting cases of disappeared or naturally expelled implants following anterior cervical spine surgery. A detailed electronic search was conducted in the PubMed database for studies published up to November 2024, using the Medical Subject Headings (MeSH) terms "anterior cervical spine surgery" and "missing/disappeared/migration into gastrointestinal tract/oral extrusion of a/an implant/hardware/screw/plate". Articles were excluded if they involved surgical intervention for esophageal perforation, lacked sufficient data on patient demographics and symptoms, resulted in mortality, or reported extrusion occurring within the first 30 days postoperatively. Studies addressing surgical interventions for abscesses or failed hardware were included. A summary table (Table 1) was prepared, incorporating the findings from 18 articles, affording the study criteria, published between 2000 and 2024, and the presented case [2,3,9-24]. 

**Table 1:** A summary of the relevant case reports ACDF - anterior cervical discectomy and fusion; ACP - anterior cervical plate; BG - bone graft; AOSF - anterior odontoid screw fixation; PEEK - polyetheretherketone; ACPR - anterior cervical plate removal; PS - posterior stabilization; N/A - not available

Author	Age/sex	Surgical procedure of the cervical spine	Implanted hardware	Symptoms before implant excretion (duration)	Time from surgery to implant excretion	Excreted implant	Radiographic/ endoscopic examination of the esophagus	Additional surgery or complications	Conservative treatment	Time to return to oral intake
											Na D, et al. [10]	61/F	ACDF	Interbody fusion device C4-5	Asymtomatic	6 months	A screw coming out of the mouth	No signs of perforation	-	N/A	N/A
Prusick PJ, et al. [11]	51/F	ACDF	Interbody fusion device C2-3, C3-4	Dysphagia (2 years)	2 years	C2-3 entire implant coming out of the mouth	N/A	-	+	N/A											
											Sath S, et al. [15]	65/M	C5 corpectomy	C5 mesh cage + ACP C4-6	Activity aggravated neck pain (1 month) + dysphagia (3 days)	2,5 months	Missing screw on radiography	N/A	Endoscopic removal of the screw (abdomen) + ACPR + PS	N/A	N/A
Yeh MY, et al. [21]	63/M	C6 corpectomy	C6 mesh cage + ACP C5-7 + PS (3 months later ACPR)	Asymtomatic	18 months (from ACPR)	Missing corpectomy cage on radiography	﻿An esophageal diverticulum	-	N/A	N/A											
											Quadri SA, et al. [2]	81/F	ACDF	﻿PEEK cage + ACP C2-3	Coughing + sense of foreign body in the throat (couple of weeks)	3,5 years	The entire implant coming out of the mouth	﻿Zenker's diverticulum	-	﻿+	5 weeks
																						Leitner L, et al. [22]	78/M	AOSF	Odontoid screw	Dysphagia (4 weeks)	8 years	Intestinal excretion of the odontoid screw	N/A	-	N/A	N/A
Duransoy YK, et al. [17]	23/M	ACDF	BG + ACP C5-6, C6-7	Hemoptiysis + dysphagia (N/A)	10 months	Missing screw on radiography	No signs of perforation	ACPR	N/A	N/A																						
											Salis G, et al. [19]	65/F	ACDF	ACP C5-6	Odinophagia + sense of foreign body in the throat (1 week)	3 years	Visible screw in the postcricoid area at the laryngoscopy	A small fistula	Laryngoscopic removal of the screw	+	6 days
Kapu R, et al. [18]	54/M	C3 corpectomy	C3 mesh cage + ACP C2-4	Dysphagia (2 months)	8 years	A screw coming out of the mouth	N/A	ACPR	﻿+	3 weeks											
											Gazzeri R, et al. [9]	45/M	C4 and C5 corpectomy	BG + ACP C3-6	Dysphagia (5 days)	11 years	﻿Missing screw on radiography	No signs of perforation	Abscess drainage + spondylodiscitis	+	7 days
Lee JS, et al. [16]	68/M	C5 corpectomy	C5 mesh cage + ACP C4-6	Sense of foreign body in the throat (2 days)	15 months	A screw coming out of the mouth	No signs of perforation	-	-	N/A											
											Martinez-Lange JF, et al. [20]	51/M	ACDF	BG + ACP C5-7	Dysphagia (N/A)	6 years	Missing screw on radiography	N/A	-	N/A	N/A
Fountas KN, et al. [13]	70/M	ACDF	﻿BG + ACP C5-6	Dysphagia (2 days)	16 months	Missing screw on radiography	Minor leakage	Endoscopic removal of the screw (abdomen) + ACPR	+	5 days											
											Wong DT, et al. [23]	56/M	C4 and C5 corpectomy	BG + ACP C3-6	﻿Upper airway obstruction	4 years	Missing screw on radiography	No signs of perforation	Abscess drainage + ACPR	N/A	N/A
Pompili A, et al. [3]	67/M	ACDF	BG + ACP C4-7	﻿ Asymptomatic	18 months	Missing screw on radiography	﻿No signs of perforation	-	N/A	N/A											
											Geyer TE, et al. [12]	76/F	ACDF	ACP C3-5	Dysphagia (3 weeks)	5 years	A screw coming out of the mouth	N/A	-	N/A	1 day
Sharma RR, et al. [14]	32/F	ACDF	BG + fixation pin C2-3	Dysphagia (N/A)	1 year	Graft and pin coming out of the mouth	N/A	-	N/A	N/A											
											Fujibayashi S, et al. [24]	67/M	ACDF	BG + ACP C7-T2	Meningitis + poor general condition (1 month)	5 weeks	Missing plate and screw on radiography	No signs of perforation	Abscess	N/A	N/A
Present case	40/M	ACDF	Anterior cervical plate C6-T1	Dysphagia (1 month)	5 years	Intestinal excretion of plate and screw	Minor leakage	Abscess	+	2 weeks											

The use of implants in anterior cervical spinal surgery offers several advantages, including rigid fixation, maintenance of proper cervical lordosis, and enhanced fusion. Additionally, implants improve the patient's quality of life by reducing the need for external fixators. Due to these benefits, implants are frequently preferred in anterior cervical spine procedures [17,18,25,26]. Despite their advantages, attention to potential hardware failure during long-term follow-up is crucial. A systematic review by Yee et al. reported a pooled incidence rate of graft or hardware failure in anterior cervical spinal surgery at 2.1%, ranging from 0% to 50% [1]. Hardware failure can result from screw malposition, poor bone quality, incomplete fusion, or posterior ligamentous instability. Furthermore, the consensus is that initial screw malposition and inadequate fixation are primary contributors to hardware failure [2,12-14,27].

Extrusion of the implanted instrumentation leading to esophageal perforation is undoubtedly one of the most severe complications. The incidence of esophageal perforation, a rare but serious complication of anterior cervical spinal surgery, ranges from 0.02% to 1.62% in the literature, with a pooled incidence rate of 0.2% reported in the review by Yee et al [1]. Abboud et al. have reported the life-threatening complication rates of 0.2-1.62% in cervical fusion surgery [28]. Although uncommon, esophageal perforation is strongly associated with increased mortality (3.92-33%) [1,4,5,7,8]. While it may be identified intraoperatively, esophageal perforation can also be diagnosed days, months, or even years after surgery. The underlying etiology and clinical presentation may vary depending on the timing of the diagnosis. When the perforation occurs within the first 30 days after surgery, it is classified as early esophageal perforation, typically presenting with acute inflammatory symptoms such as sepsis, mediastinitis, abscess, and cervical swelling. Perforations diagnosed after 30 days are considered delayed, with dysphagia and odynophagia as the primary symptoms. Most early perforations are iatrogenic, resulting from improper positioning or movement of the retractor blades during surgery. In contrast, delayed perforations are typically due to microtrauma and chronic erosion caused by hardware failure or implant compression [4,8,11,29]. Even in patients with a long history of anterior cervical spinal surgery, it is essential for clinicians to promptly recognize symptoms such as dysphagia and odynophagia and initiate relevant investigations, as early diagnosis and treatment are critical for managing this potentially fatal complication. CT is an easily accessible imaging modality that evaluates the area around the esophagus and implant, helping identify extraluminal air or abscesses. Additionally, contrast esophagography and endoscopy play key roles in the diagnostic process [2,4,7,30].

The primary treatment for esophageal perforations is surgical repair, often accompanied by the revision or removal of the causative implant. While primary closure may suffice in certain cases, repair of the defect using a muscle flap, a complex surgical procedure, is usually preferred [7,8,29,30]. This approach is usually effective in early-detected and uncontaminated cases. If enough time had passed over the esophageal perforation as to cause periesophageal contamination, it would be quite difficult to repair such an esophageal defect, even though there are no signs of sepsis. Periesophageal infection makes every type of esophageal repair hazardous. However, a single surgical procedure is not always adequate for managing this challenging condition. In a systematic review by Halani et al., the average number of surgeries per patient for esophageal perforation was reported as 1.54 [8].

Another peril in dislodged anterior cervical implants is the embedded or indwelled implants through the esophageal wall, which are caught just passing into the esophageal lumen. Removal of the implant in this stage of its journey may result in jeopardy without giving the opportunity for the healthy repair of the esophageal wall defect. In such conditions, the most judicious way is to react in a conservative way. We propose to manage the dislodged implants that are dropped into the gastrointestinal tube lumen or just transiting the wall of the tube with a "watch and wait" approach. Unless the patient suffers from disphagia and/or odinophagia that restricts oral food intake, surgery needs to be avoided. During this period, clinical and radiological follow-up should be continued. Even though the surgical intervention is decided, a last-minute (early in the morning of the operation) radiologic image should be taken before the operation. Conservative treatment primarily involves cessation of oral intake and administration of intravenous antibiotics, relying on the spontaneous healing of minor defects. This approach is the least practiced option in treating esophageal perforation due to its high association with mortality. Its adoption requires strict criteria, including a minor or absent fistula, mild or no infection, and a defect size of less than 1 cm [4,6,12,13,27]. In some instances, however, the patient's poor overall condition may necessitate conservative management as the only viable option [24]. Particularly in dramatic cases where an implanted instrument has extruded and been excreted via the oral or gastrointestinal tract, a minor esophageal defect and an uncomplicated treatment course are unexpected.

Nevertheless, there are rare exceptional case reports in the literature where such cases were successfully managed without aggressive surgical intervention [2,3,9-22]. Harman et al. outlined a treatment algorithm for esophageal perforations after anterior cervical surgery, but did not address the importance of last-minute imaging and the efficacy of conservative treatment in cases of naturally excreted or missing implants [7]. The present study reviews case reports of implant migration leading to delayed pharyngoesophageal perforation after anterior cervical surgery, managed without surgical intervention (see Table 1). Therefore, the potential for migration must always be considered when dealing with dislodged implants. As demonstrated in the present case, even recent imaging may not reflect the current location of the implant, emphasizing the need for last-minute imaging before any surgical procedure. 

Although dysphagia is the most common presenting symptom in the reviewed cases, clinical manifestations can vary widely, from being asymptomatic to experiencing upper airway obstruction or even oral expulsion of the implant. While symptoms typically last 1-2 weeks in most cases, milder presentations may persist for years, with patients tolerating significant discomfort. The timeframe for implant excretion after anterior cervical surgery ranges from five weeks to 11 years, with the majority of cases occurring more than a year post-surgery. That underscores the importance of long-term follow-up in patients with surgically implanted devices.

The concept that the slow migration process of an implant into the esophagus may allow the tissue to repair itself, allowing the defect to heal spontaneously or to be small in size, has been previously mentioned by Pompili et al. [3]. Limiting the size of the fistula may reduce the risk of septic complications. Furthermore, the absence of fistula and infection in most cases included in the review emphasizes the effectiveness of slow migration and tissue repair mechanisms. Conversely, migrating larger implants into the esophagus may result in complications such as abscesses or diverticula [2,21,24]. Abscess formation, particularly concerning due to its proximity to the airway and vital vessels, can often be managed with antibiotic therapy once the implant has migrated away from the affected area, as demonstrated in the present case. Upper gastrointestinal endoscopy may be useful to evaluate the size of the fistula and implant position through its way - how far indwelled into the esophagus wall. Cianci et al. suggest that 80-90% of gastrointestinal foreign bodies pass naturally and simply through the digestive tract, but a significant amount impacts the upper aerodigestive tract [31]. Complications associated with foreign body impaction include ulcers (21.2%), lacerations (14.9%), erosion (12%), and perforation (1.9%) [32]. Although the treatment of choice is the endoscopic retrieval in these cases, 10-12% require endoscopic extraction, and only 1% need surgery [32,33].

Liu et al., in accordance with the European Society for Gastrointestinal Endoscopy (ESGE) guidelines, recommend that endoscopic removal of ingested esophageal foreign bodies that obstruct the lumen should be extracted within six hours to reduce the complication rate in an emergency setting [34]. ESGE recommends emergent endoscopy within six hours and urgent endoscopy within 24 hours for different kinds of foreign objects in the esophagus [35]. In our patient, we did not consider upper GI endoscopy unless we detected the symptoms of esophageal obstruction and followed the patient conservatively with sequential imaging studies. In the systematic review by Halani et al., the group of patients treated conservatively was younger (median age 37.3 years), and it was noted that youth may offer enhanced wound healing capacity [8]. However, in the present study, the median age of patients with delayed esophageal perforation secondary to implant excretion who recovered without surgery was 58.5 years (ranging from 23 to 81 years). Elderly patients, in particular, may endure or overlook symptoms such as dysphagia, leading to a prolonged course that may contribute to slow migration. The time to return to oral intake is a significant indicator of treatment success. Halani et al. reported that the time to return to oral intake in the conservatively treated group (mean 68 days) was remarkably longer than in the surgically treated group (mean 28 days) [8]. Among the studies that included information on the time to return to oral intake, the mean duration was 12.7 days (ranging from one day to 5 weeks) [2,9,12,13,18,19].

Implants above the C3 level are more likely to cause damage to the posterior wall of the pharynx, and excretion of the implant may occur via the oral route [2,10,11,18]. An intriguing observation from the review was that oral implant excretion was more prevalent in female patients. In contrast, excretion or loss through the gastrointestinal tract was more common in male patients. The study by Leitner et al. and the presented case demonstrate that implants that migrate into the gastrointestinal tract can be excreted through the stool [22].

Asymptomatic elimination via the gastrointestinal tract is also probable in articles reporting missing implants [3,9,17,20-24]. In the majority of published case reports, the excreted or lost material is typically a small screw or pin. A grave scenario is expected when the entire implant extrudes into the pharyngoesophageal area [36]. The case reports presenting the contrary progress are exceedingly rare [2,11,21,22]. The presented case is unique in that the journey of the anterior cervical plate through the gastrointestinal tract is well-documented radiologically, with a last-minute notice of migration prior to surgery. Early diagnosis and appropriate treatment for this complication are the key to reducing morbidity and mortality, as the main prognostic factor is the interval between the onset of the fistula, diagnosis, and treatment.

## Conclusions

This study highlights the following key points. Long-term follow-up of patients undergoing anterior cervical surgery and awareness of potential complications are crucial. Last-minute radiological imaging is critical in patients with implant dislodgement who are followed up conservatively, when surgery is planned. Esophageal perforation is a severe complication associated with high mortality, and surgical intervention remains the primary treatment modality. Nevertheless, a carefully selected subset of patients may be managed with "watch-and-wait" conservative approach under close clinical surveillance. It is conceivable that the missing hardware may have been naturally excreted through the gastrointestinal tract without causing gastrointestinal symptoms and even need a gastrointestinal endoscopy.
